# Migraine headaches in Chronic Fatigue Syndrome (CFS): Comparison of two prospective cross-sectional studies

**DOI:** 10.1186/1471-2377-11-30

**Published:** 2011-03-05

**Authors:** Murugan K Ravindran, Yin Zheng, Christian Timbol, Samantha J Merck, James N Baraniuk

**Affiliations:** 1 Division of Rheumatology, Immunology and Allergy, Room 3004F, 3rd Floor PHC Building, Georgetown University, 3800 Reservoir Road, NW, Washington, DC 20007-2197 USA

## Abstract

**Background:**

Headaches are more frequent in Chronic Fatigue Syndrome (CFS) than healthy control (HC) subjects. The 2004 International Headache Society (IHS) criteria were used to define CFS headache phenotypes.

**Methods:**

Subjects in Cohort 1 (HC = 368; CFS = 203) completed questionnaires about many diverse symptoms by giving nominal (yes/no) answers. Cohort 2 (HC = 21; CFS = 67) had more focused evaluations. They scored symptom severities on 0 to 4 anchored ordinal scales, and had structured headache evaluations. All subjects had history and physical examinations; assessments for exclusion criteria; questionnaires about CFS related symptoms (0 to 4 scale), Multidimensional Fatigue Inventory (MFI) and Medical Outcome Survey Short Form 36 (MOS SF-36).

**Results:**

Demographics, trends for the number of diffuse "functional" symptoms present, and severity of CFS case designation criteria symptoms were equivalent between CFS subjects in Cohorts 1 and 2. HC had significantly fewer symptoms, lower MFI and higher SF-36 domain scores than CFS in both cohorts. Migraine headaches were found in 84%, and tension-type headaches in 81% of Cohort 2 CFS. This compared to 5% and 45%, respectively, in HC. The CFS group had migraine without aura (60%; MO; CFS+MO), with aura (24%; CFS+MA), tension headaches only (12%), or no headaches (4%). Co-morbid tension and migraine headaches were found in 67% of CFS. CFS+MA had higher severity scores than CFS+MO for the sum of scores for poor memory, dizziness, balance, and numbness ("Neuro-construct", p = 0.002) and perceived heart rhythm disturbances, palpitations and noncardiac chest pain ("Cardio-construct"; p = 0.045, t-tests after Bonferroni corrections). CFS+MO subjects had lower pressure-induced pain thresholds (2.36 kg [1.95-2.78; 95% C.I.] n = 40) and a higher prevalence of fibromyalgia (47%; 1990 criteria) compared to HC (5.23 kg [3.95-6.52] n = 20; and 0%, respectively). Sumatriptan was beneficial for 13 out of 14 newly diagnosed CFS migraine subjects.

**Conclusions:**

CFS subjects had higher prevalences of MO and MA than HC, suggesting that mechanisms of migraine pathogenesis such as central sensitization may contribute to CFS pathophysiology.

**Clinical Trial Registration:**

Georgetown University IRB # 2006-481

ClinicalTrials.gov NCT00810329

## Background

Headaches are common complaints in Chronic Fatigue Syndrome (CFS) and fibromyalgia (FM) [1,2]. However, the characteristics of these headaches are poorly defined with regards to the International Headache Society criteria (IHS) [3].

The CFS case designation requires a 6 month history of persistent, unexplained fatigue that causes significant impairment of activity, plus 4 of 8 associated criteria [1]. These criteria include exertional exhaustion, cognitive and sleep disturbances, and diffuse bodily pain including headaches. Fatigue was verified using a 5 point anchored ordinal scale [4] and the Multidimensional Fatigue Inventory [5]. Disability and impaired quality of life were confirmed using the Medical Outcomes Survey Short Form 36 (SF-36) [6]. Headache severity was assessed by a 5 point ordinal scale [4]. Pain symptoms were compared between groups using the McGill Pain Short Form [7]. Acute and chronic medical, surgical and psychiatric conditions were ruled out by history and physical examinations.

Headaches and other CFS related symptoms overlap with fibromyalgia (FM) criteria. The 1990 American College of Rheumatology (ACR) research definition of FM required 3 months of unexplained, widespread pain plus tenderness to manual thumb pressure at ≥ 11 of 18 traditional tender points [8]. This definition has been replaced by the 2010 FM criteria [9] that emphasize subjective assessments of widespread pain, fatigue, unrefreshing sleep, and cognitive difficulties. The new criteria allow considerable overlap between CFS and FM conditions.

The most prevalent headaches in the general population are tension - type (45%), migraine (12%), sinus (3%), and medication overuse headaches (1 - 2%) [3,10,11]. Tension - type (T) headaches are defined by bilateral, pressing or nonpulsatile cephalgia of mild to moderate intensity that may last 30 minutes to 7 days. They are not aggravated by routine physical activity such as walking or stair climbing.

Migraine is diagnosed by having at least 5 episodes lasting 4 to 72 hr with at least 2 of the following criteria: (i) unilateral location; (ii) pulsatile quality; (iii) moderate to severe pain intensity; and (iv) aggravation by physical activities or active avoidance of movement. Either nausea ± emesis, or photophobia and phonophobia must occur. Migraines are subdivided into migraine with aura (MA; 20% to 30% of migraineurs) and migraine without aura (MO) [3,12,13]. The focal sensory phenomena of the aura involves either homonymous visual symptoms such as flickering lights, rotating discs, photosensitivity, and loss of vision, or unilateral somatosensory disturbances such as paresthesias or numbness. Symptoms appear gradually over 5 to 20 minutes and last less than 60 minutes. The aura must be detected at least twice. Motor function is not affected. The headache phase of the migraine attack usually begins within 60 minutes after the end of the aura, but sometimes is delayed for several hours. In some cases, pain can be completely absent. Multiple avenues of investigation including brain magnetic resonance imaging (MRI) techniques have associated auras with the concentric expansion of severe and prolonged brain cortex electrical depolarization, neurotransmitter release, vasoconstriction and reactive hyperemia known as cortical spreading depression [14]. Increased pain sensitivity (hyperalgesia) may occur in both MO and MA [15] due to dysfunctional pain regulatory mechanisms during central sensitization [16].

Since 67% of migraine subjects meet CFS criteria [2], we propose that mechanisms of migraine may play roles in CFS pathophysiology. If so, CFS subjects should have elevated rates of migraine headaches compared to control subjects. Mechanisms that differentiate MO from MA may also be present and permit subgroup analysis in CFS.

## Methods

Two cohorts of CFS [1] and healthy control (HC) subjects were recruited in sequential order (Cohort 1 then Cohort 2) using identical advertisements in the community (e.g. newspapers) and the tertiary medical center (e.g. posters), and information from websites [17,18]. All subjects gave signed informed consent to take part in this IRB - approved study.

History and physical examinations assessed the CFS case designation criteria. "CFS" was designated by findings of significant, unexplained fatigue lasting at least 6 months plus the presence of at least 4 of 8 ancillary criteria: (i) problems with concentration or memory; (ii) sleep disturbances; (iii) exertional exhaustion that typically developed 24 hr after excessive physical or mental effort; (iv) muscle pain; (v) joint pain; (vi) headaches; (vii) sore throat; and (viii) sore lymph node regions in the cervical, axillary or inguinal areas [1]. These CFS symptoms were scored in nominal fashion as "present" or "absent". In addition, subjects scored the severity of each symptom in the previous 6 months using a 5 point, anchored ordinal scale [4]. Scores were 0 for no symptom, 1 for trivial, 2 for mild, 3 for moderate and 4 for severe complaints. Inclusion of "trivial" allowed subjects to acknowledge complaints that were present but not bothersome enough to warrant treatment or other lifestyle adaptations [19]. Subjects with untreated endocrine, major psychiatric, cardiovascular, infectious (e.g. HIV), neoplastic, and other chronic diseases that may have accounted for fatigue, body pain, headache or other symptoms were excluded.

Cohort 1 was assessed by a series of questionnaires, history and physical examinations. Different combinations of questionnaires were used during their recruitment period. Preliminary results from Cohort 1 suggested an increased prevalence of migraine in CFS compared to HC.

Next, Cohort 2 was recruited. Subjects completed an optimized set of questionnaires and had formalized clinical interviews to diagnose migraine with (MA) and without (MO) aura, and tension - type (T) headaches based on IHS criteria (as outlined in the Introduction) [3]. CFS subjects were classified as CFS+MA, CFS+MO, and CFS+T, respectively. About two - thirds of CFS+MA and CFS+MO subjects had co-morbid tension-type headaches. For statistical convenience, the CFS+T group included 3 subjects with no headaches.

The Multidimensional Fatigue Inventory was used to verify the presence of significant physical, mental, and other fatigue measures [5]. Quality of life (disability) was assessed by the Medical Outcomes Survey Short Form 36 (SF-36) domain scores [6]. Pain complaints were subjectively assessed using the McGill Pain Short Form with its Affective, Sensory and Total Scores [7]. Subjects completed a multisystem complaints questionnaire that evaluated the nominal presence or absence of migraine and tension headaches, musculoskeletal, airway, thoracic, bowel, bladder and other symptoms over the previous 3 months [4].

The frequencies of several symptoms from individual systems were found to be highly correlated with each other in preliminary studies. Therefore, questionnaires were modified to also score the severities of each symptom. The same 0 to 4 point, anchored, ordinal scale was used, and data collected for some Cohort 1 and all Cohort 2 subjects. The severity scores for the selected symptoms were again found to correlate with each other and the Fatigue Severity Score. The sum of severity scores for numbness in the arms and legs, problems with memory, dizziness and balance was calculated and accounted for 32% of the explained variance (R^2^) between these queries and the Fatigue Score. This sum was defined as the "Neuro-construct" (score range of 0 to 16). A "Cardio-construct" was developed as the sum of scores for rapid heart rate, irregular heart rate, palpitations, and chest pain (range 0 to 16). The explained variance was 30%.

FM was assessed using the 1990 American College of Rheumatology (ACR) research definition. Diagnosis of FM required 3 months of unexplained, widespread pain affecting all 4 quadrants of the body and the axial skeleton, plus tenderness to manual thumb pressure (~4 kg) at ≥ 11 of 18 traditional tender points [8]. Pressure - induced pain thresholds were measured by pushing a strain gauge tipped with a 1cm^2 ^rubber tip (dolorimeter) at a rate of 1 kg/sec on the same 18 anatomical sites. The average was the systemic pain threshold (kg). The pressure required to cause pain was also measured over the frontal, ethmoid and maxillary paranasal sinus regions. The average was the sinus pain threshold [20].

Means and 95% confidence intervals were calculated for each variable in the HC and CFS groups [21,22]. Cohort 1 HC and CFS outcomes were compared using 2-tailed unpaired Student's t-tests with Bonferroni corrections for multiple comparisons (p × 138 comparitors) to identify significant differences. Fisher's Exact tests evaluated differences in proportions [21]. Significance was ascribed to p ≤ 0.05.

The Cohort 2 HC and CFS and headache subtype data were compared by analysis of variance (ANOVA). If significant, variables were compared by 2-tailed unpaired Student's t-tests with Bonferroni corrections. Fisher's Exact tests were used as appropriate.

## Results

### Demographics, Disability, and Fatigue

There were no significant differences in age or gender distribution between HC and CFS subjects in either Cohort 1 or Cohort 2 after Bonferroni corrections of p values (Table 1). There were no significant differences of age, gender, or body mass index (BMI) for Cohort 2 HC and the CFS plus headache subtypes.

**Table 1:** Demographics for Cohorts 1 and 2 (mean [95% confidence intervals]; and number with percentage).

	Cohort 1		Cohort 2				
	HC	CFS	HC	CFS	CFS+MO	CFS+MA	CFS+T
N	368	203	21	67	40	16	11
Age	41.1 [39.7-42.5]	44.6 [42.9-46.2]	42.1 [36.3-47.9]	46.1 [43.7-48.6]	45.4 [42.3-48.5]	46.5 [42.4-50.6]	48.4 [40.5-56.2]
Male	85 (23%)	244 (12%)	13 (62%)	23 (34%)	11 (28%)	6 (38%)	6 (55%)
BMI	-	-	27.6 [25.0-30.2]	29.3 [26.7-30.8]	29.2 [27.1-31.3]	29.4 [25.7-33.1]	29.7 [24.2-33.3]
							
Course of CFS		Rapid onset (< 1 month)		48 (71%)	32 (81%)	14 (87%)	3 (23%)
		Gradually progressive		19 (29%)	8 (19%)	2 (13%)	8 (77%)

Most of the CFS subjects from both cohorts had rapid onset of CFS symptoms following an initiating event. Twenty-three of the Cohort 2 CFS subjects reported motor vehicle accidents, head trauma, or concussions within 5 years of the onset of their migraines (34%). Five felt they had severe flu-like illnesses that never improved. Three gave histories of prolonged, high dose exposures to volatile organic compounds, and two stated symptoms began soon after extensive series of immunizations. Three had perimenstrual migraines that predated their CFS.

The HC groups within both cohorts had significantly better quality of life (higher scores) for all domains of the SF-36 compared to CFS subjects (Table 2). Role Emotional and Mental Health domains for the Cohort 2 CFS headache subgroups were not as significantly different from HC (0.05 > p > 0.03) as for the other domains (p ≤ 0.0003). The Multidimensional Fatigue Inventory domains were significantly higher in CFS than HC in both cohorts, and for the 3 CFS plus headache subgroups in Cohort 2 (Table 3). These results indicated that the two cohorts of control and CFS subjects were comparable to each other.

**Table 2:** MOS-SF-36 Quality of Life Domains for Cohorts 1 and 2 (mean [95% confidence intervals]; probability).

	Cohort 1		Cohort 2				
Domain	HC n = 295	CFS * n = 145	HC n = 19	CFS ** n = 49	CFS+MO** n = 28	CFS+MA** n = 12	CFS+T ** n = 9
Physical Functioning	71.6 [67.8-75.4]	47.6 [43.4-52.1] p < 10^-10^	91.1 [82.4-99.7]	41.4 [34.6-48.2] p < 10^-10^	43.7 [35.5-51.9] p = 10^-9^	28.8 [15.6-41.9] p = 10^-8^	51.7 [34.5-68.8] p = 0.0001
Social Functioning	67.4 [63.2-71.6]	46.4 [41.8-51.0] p = 10^-9^	87.5 [76.5-98.5]	26.9 [21.0-32.8] p < 10^-10^	25.5 [17.9-33.0] p < 10^-10^	23.9 [9.3-38.4] p = 10^-7^	35.9 [26.2-45.7] P = 0.000005
Role-Physical	60.5 [63.2-71.6]	18.5 [13.3-23.7] p < 10^-10^	76.3 [57.8-94.9]	5.6 [0.8-10.4] p < 10^-10^	8.0 [0.5-15.6] p = 10^-9^	4.2 [-4.0-12.3] p = 10^-6^	0 P = 0.000009
Role-Emotional	71.6 [62.8-80.3]	53.3 [46.2-60.5] p = 0.008	86.0 [72.5-99.5]	57.6 [44.8-70.5]	59.3 [42.0-76.5] p = 0.03	55.6 [28.5-82.7] p = 0.04	55.6 [24.8-86.4] p = 0.048
Mental Health	66.8 [63.0-70.5]	58.4 [54.9-61.9] p = 0.006	79.2 [74.0-84.3]	62.6 [57.0-68.3]	64.9 [57.9-71.8] p = 0.005	59.7 [45.1-74.2] p = 0.007	59.6 [48.1-71.0] p = 0.001
Vitality	50.1 [47.1-53.1]	25.4 [22.3-28.5] p < 10^-10^	66.1 [56.5-75.8]	13.9 [10.5-17.2] p < 10^-10^	14.8 [10.3-19.3] p < 10^-10^	6.7 [2.1-11.2] p = 10^-10^	20.6 [12.6-28.5] p = 10^-6^
Bodily Pain	60.5 [56.5-64.6]	35.1 [31.4-38.8] p < 10^-10^	83.8 [72.4-95.2]	34.1 [28.1-40.2] p < 10^-10^	32.8 [25.3-40.3] p = 10^-9^	30.6 [16.2-44.9] p = 10^-6^	42.8 [29.5-56.0] p = 0.0003
General Health	64.5 [56.5-64.6]	38.3 [34.3-42.3] p < 10^-10^	76.8 [69.5-84.2]	33.0 [27.8-38.2] p < 10^-10^	36.2 [28.3-44.1] p = 10^-8^	28.8 [19.9-37.6] p = 10^-8^	29.1 [20.9-37.4] p = 10^-8^

2-tailed unpaired Student's t-tests with Bonferroni corrections following ANOVA (p < 0.05): * vs. HC of Cohort 1 and ** vs. HC of Cohort 2.

**Table 3:** Multidimensional Fatigue Inventory Domains (mean [95% confidence intervals]; probability).

	Cohort 1		Cohort 2				
Domains	HC n = 300	CFS * n = 170	HC n = 19	CFS ** N = 49	CFS+MO** n = 28	CFS+MA** N = 12	CFS+T ** n = 9
General Fatigue	10.4 [9.9-10.9]	16.4 [15.9-16.9] p < 10^-10^	8.2 [6.7-9.6]	17.9 [17.2-18.6] p < 10^-10^	18.3 [17.5-19.1] p < 10^-10^	18.5 [17.0-20.0] p = 10^-10^	15.9 [13.9-17.8] p = 10^-6^
Physical Fatigue	9.4 [8.9-9.9]	14.6 [14.0-15.2] p < 10^-10^	8.1 [5.8-10.3]	16.0 [15.1-16.9] p < 10^-10^	16.3 [15.1-17.5] p = 10^-8^	16.3 [14.6-17.9] p = 0.00002	14.8 [12.2-16.5] p = 0.002
Reduced Activity	8.4 [8.0-8.8]	12.7 [12.1-13.4] p < 10^-10^	7.4 [5.7-9.2]	16.2 [15.2-17.1] p < 10^-10^	16.3 [15.0-17.5] p < 10^-10^	17.4 [15.8-19.1] p = 10^-8^	14.3 [12.2-16.5] p = 0.00001
Reduced Motivation	8.2 [7.6-8.7]	11.1 [10.5-11.7] p < 10^-10^	8.2 [6.7-9.7]	11.9 [10.7-13.1] P = 0.001	11.8 [10.1-13.4] p = 0.005	13.0 [10.3-15.7] p = 0.003	10.7 [8.8-12.5]
Mental Fatigue	8.8 [8.3-9.3]	13.2 [12.5-13.9] p < 10^-10^	8.5 [6.9-10.1]	15.0 [13.8-16.1] P = 10^-8^	14.7 [13.2-16.1] p = 10^-6^	16.8 [14.7-18.8] p = 10^-6^	13.3 [10.4-16.2] p = 0.004

2-tailed unpaired Student's t-tests with Bonferroni corrections following ANOVA (p < 0.05): * vs. HC of Cohort 1 and ** vs. HC of Cohort 2.

### Headaches

Headache types were first assessed by self - report questionnaires. HC reported migraines in 12% (Cohort 1) and 16% (Cohort 2), and tension headaches in 22% and 28%, respectively. Migraines were reported to be more prevalent in the Cohort 2 CFS group (69%) compared to Cohort 1 (39%). The point prevalence rates for tension - type headaches were not significantly different between Cohort 1 (62%) and 2 (76%) CFS groups. The combination of migraine plus tension headaches was claimed to be present in 3% of HC and 61% of CFS in Cohort 1. This combination was present in 11% of HC and 60% of CFS in Cohort 2.

Cohort 2 subjects were interviewed using IHS criteria to determine their headache type. Point prevalences in HC (n = 21) were 16% migraine, 28% tension and 11% with both. In contrast to questionnaires, interviews of these HC subjects identified migraine in 5%, and tension headaches in 45%. Interviews of 67 CFS subjects identified migraine in 84%, tension headaches in 81%, and both types in 67%. MO was found in 60% (40/67), and MA in 24% (16/67). Two CFS subjects had migraines that were intermittently associated with auras. Tension headaches were present in 78% (31/40) of MO and 88% (14/16) of MA. The CFS+T group (n = 11) included 3 with no headaches.

CFS subjects attributed the high prevalences of headaches to their impression that head pain was a usual and often daily manifestation of the severe fatigue and general pain syndrome they experienced. They were often unaware of MO headaches and the potential to use beneficial migraine treatments. Nonsteroidal anti-inflammatory drugs were used by all subjects, raising the possibility of medication overuse headaches as a complicating factor in CFS.

Migraine frequency and treatment information were obtained from 51 of the 67 CFS subjects. Headaches occurred on a yearly to monthly basis in 27 subjects (53%). Twenty of these had been previously diagnosed, and 7 were treated with a triptan drug. Half of this subset (10/20) had no specific headache evaluation or treatment plan. Three subjects were symptomatic with migraine during their interviews, and received new diagnoses at that time. All 3 responded to sumatriptan.

Headaches were present for 1 to 7 days per week in 47% of the 51 CFS migraine subjects. Only 14 of these 24 had been previously diagnosed, and only 3 were prescribed triptan drugs. Of the 11 with active headaches during the interviews that led to new migraine diagnoses, 10 had beneficial responses to sumatriptan within 12 hours. In total, 13 out of 14 newly diagnosed migraine subjects responded to sumatriptan. This indicates that diagnosis of migraine and its treatment with triptans was of significant benefit in CFS subjects. Migraines were under diagnosed and under treated in CFS subjects.

### CFS Criteria

**Figure 1:**
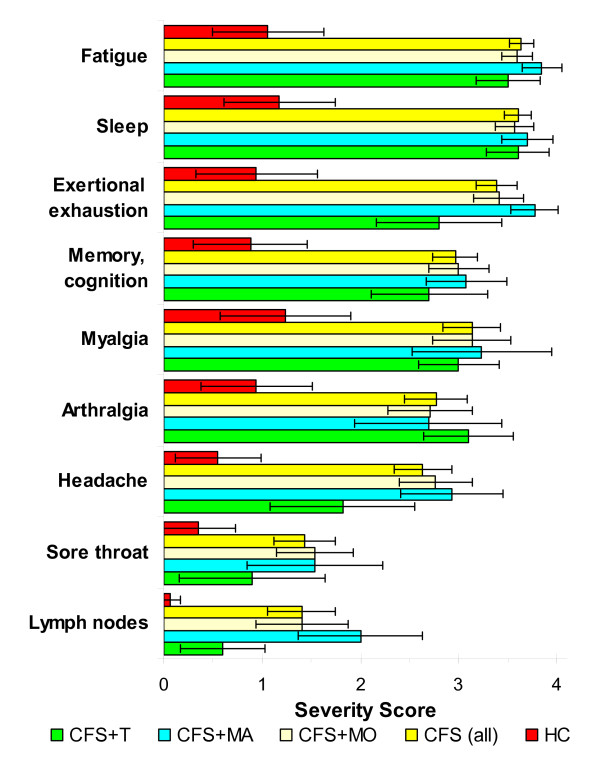
**CFS symptom severity scores for Cohort 2**. Symptom severities were scored on ordinal scales from 0 to 4. HC (red columns) had significantly lower scores for each item (mean; 95% C.I. error bars) compared to all CFS (yellow columns) (p ≤ 0.006 after Bonferonni corrections). HC scores were also significantly lower than all the CFS headache subtypes and items except sore throat in CFS+T (green bar). CFS+MA (blue columns) had significantly higher scores than CFS+T for exertional exhaustion (p = 0.036) and sore lymph nodes (p = 0.017).

The severity scores for each of the 9 CFS criteria (range 0 to 4 for each item) were compiled to examine differences based on headache subtypes. Fatigue Severity Scores were significantly lower in HC than each of the CFS headache subgroups and the composite group of all CFS subjects (p < 10^-7 ^by 2-tailed unpaired Student's t-tests with Bonferroni corrections following significant ANOVA) (Figure 1). CFS and CFS headache subgroup scores were significantly higher than HC (p < 0.006) except for sore throat in CFS+MA (p = 0.02), and headache (p = 0.03) and sore lymph nodes (p = 0.04) in the CFS+T subgroup. Headache Severity Scores were significantly higher in CFS+MO, CFS+MA and total CFS groups compared to HC (p = 10^-8 ^by ANOVA; p ≤ 10^-6 ^for each comparison after Bonferroni comparisons).

### Other questionnaires

McGill Pain Scores were significantly higher in the CFS than HC groups of Cohorts 1 and 2 (Table 4). Both the CFS+MO and CFS+MA subtypes had significantly higher scores than HC in Cohort 2 (p = 0.0027 by ANOVA and p < 0.007 by t-tests). These differences were driven by the Sensory Subscale. "Neuro-construct" scores were highest in CFS+MA (11.4 out of 16) (Table 4). CFS+MA scores were significantly higher than HC (p = 10^-8^), CFS+MO (p = 0.002) and CFS+T (p = 0.00002; t-tests). "Cardio-construct" scores were significantly higher for CFS+MA (5.9 out of 16) than CFS+MO (p = 0.045; t-tests) and CFS+T (p = 0.049). These results were consistent with the elevated scores of Cohort 1 CFS subjects (Table 4).

**Table 4:** Subjective scores that were significantly different from HC and CFS+MA (mean [95% C.I.]; probability).

	Cohort 1		Cohort 2				
	HC	CFS	HC	All CFS	CFS+MO	CFS+MA	CFS+T
Headache Severity Score	0.85 [0.69-1.01] N = 191	2.64 [2.45-2.83] n = 152 p < 10^-10 ^*	0.55 [0.11-0.99] n = 20	2.63 [2.33-2.93] n = 63 p < 10^-9 ^**	2.76 [2.39-3.13] n = 38 p < 10^-8 ^**	2.93 [2.43-3.43] n = 14 p = 10^-6 ^**	1.82 [1.08-2.56] n = 11 p = 0.03 **
McGill Sensory Score	6.5 [5.2-8.8] n = 109	8.8 [7.0-10.5] n = 55 p = 0.002 *	4.3 [0.9-7.7] n = 18	13.9 [11.5-16.3] n = 44 p = 0.0004**	13.6 [10.5-16.6] n = 24 p = 0.002**	17.5 [11.6-23.4] n = 12 p = 0.002 **	9.50 [7.0-12.0] n = 8
Neuro-Construct	2.39 [1.58-3.20] n = 57	7.00 [4.91-9.01] n = 21 p = 0.000005*	1.71 [0.36-3.06] n = 17	7.08 [5.93-8.23] n = 48 p = 0.00003**	6.33 [4.92-7.74] n = 30 p = 0.0007** p = 0.002***	11.4 [10.3-12.5] n = 10 p = 10^-8 ^**	4.50 [2.84-6.16] n = 8 p = 0.00002***
Cardio-Construct	1.46 [0.79-2.13] n = 57	2.86 [1.45-4.27] n = 21 p = 0.053	0.76 [-0.07-1.59] n = 17	3.22 [2.30-4.14] n = 45 p = 0.02**	2.70 [1.67-3.73] n = 27 p = 0.045***	5.90 [3.55-8.25] n = 10 p = 0.0004 **	1.63 [0.65-2.61] n = 8 p = 0.049 ***

* vs. HC of Cohort 1; ** vs. HC of Cohort 2; and *** vs. CFS+MA using 2-tailed unpaired Student's t-tests with Bonferroni corrections after ANOVA. Neuro-Construct = sum of scores for poor memory, dizziness, balance problems, and numbness in arms or legs. Cardio-Construct = sum of scores for rapid heart rate, irregular heart rate, palpitations, and chest pain.

### Fibromyalgia and dolorimetry

Significantly more CFS+MO (55.3%) and CFS+MA (46.2%) subjects complained of widespread pain in all four quadrants and their axial skeleton compared to HC (10.0%; p = 0.0010 by Fisher's Exact tests vs. All CFS) (Table 5). Positive tender points (≥ 11/18) were found more commonly in CFS (55.4%) than HC (5.6%; p = 0.00005). Based on the 1990 ACR criteria [4], fibromyalgia was present in 37.7% of CFS, 47.4% of CFS+MO, 28.5% of CFS+MA, and 11.1% in CFS+T, but none of the HC subjects (p = 0.0022 by ANOVA).

**Table 5:** Fibromyalgia (1990 Criteria) [8] and pressure - induced pain thresholds in Cohort 2 (mean [95% confidence intervals]; probability).

	HC	All CFS	CFS+MO	CFS+MA	CFS+T
	n = 20	N = 67	n = 40	n = 16	n = 10
Widespread pain	10.0%	50.0% P = 0.0010*	55.3% p = 0.0006*	46.2 p = 0.02*	33.3%
Manual pressure at ≥ 11/18 Points	5.6%	55.4% p = 0.00005*	64.1% p = 0.000013*	56.3% p = 0.001*	20.0% p = 0.014**
Fibromyalgia	0%	37.7% P = 0.0004*	47.4% P = 0.00007*	28.5% p = 0.022*	11.1% p = 0.03**
					
Systemic pain threshold (kg)	5.23 [3.95-6.52]	2.90 [2.47-3.32] p = 0.0002 ^†^	2.36 [1.95-2.78] P = 0.00001 ^†^	3.51 [2.53-4.48]	3.94 [2.67-5.20] p = 0.025 ^††^
Sinus pain threshold (kg)	1.91 [0.37-2.45]	p = 0.0002 ^†^	0.87 [0.71-1.03] p = 0.0001 ^†^	1.18 [0.86-1.51]	1.30 [0.90-1.71]

Fisher's Exact Test vs.* HC and vs. ** CFS+MO. 2-tailed unpaired Student's tests with Bonferonni corrections following ANOVA (p < 0.05) vs. ^† ^HC and vs. ^†† ^CFS+MO.

The average pressure causing pain at the 18 tender points was measured by dolorimetry. The CFS+MO subgroup had a significantly lower pain threshold (2.36 kg) compared to HC (5.23 kg; p = 0.00001 by Bonferroni corrected t-test and p = 0.0006 by ANOVA) (Table 5). Pain thresholds were not correlated with age or gender (data not shown). Tenderness was assessed over the facial sinus regions. CFS subjects had lower pain thresholds (1.02 kg) than HC (1.91 kg; p = 0.0002, t-test), with the CFS+MO subgroup being the most tender (0.87 kg; p = 0.00001).

## Discussion

CFS, FM and headaches were defined by personal history and physical examinations in this study rather than by surveys as reported previously [2,23,24]. The headache interview used for Cohort 2 identified the frequencies of migraine subtypes and tension headache in both CFS and HC groups. The self-report questionnaires gave comparable results for Cohorts 1 and 2. However, the questionnaire findings were not accurate when compared to the structured interviews. The interviews provided credibility to the presence of CFS+MA and CFS+T subgroups despite their small sample sizes. The interviews also demonstrated the higher rates of tension headaches in HC, and migraine without aura (MO) in CFS. CFS subjects with tension headaches (CFS+T) tended to have less severe complaints compared to the migraine subtypes. Inclusion of quality of life SF-36 and fatigue (MFI) questionnaires confirmed the disability and spectrum of fatigue manifestations experienced by the CFS groups.

These data verified the high prevalence of migraine pathology in CFS [2]. The ratio of MO to MA was similar to that reported in other migraine groups [10,12]. We propose that mechanisms of migraine pathophysiology may contribute to other CFS symptoms in addition to headaches. If so, anti - migraine treatments may be beneficial for CFS - related symptoms even in subjects who do not have migraines. This speculation requires prospective evaluation in clinical trials.

Correlates of MO and MA suggested that aura may be associated with distinct phenotypic and pathological alterations. Neuro-construct, Cardio-construct, and McGill Pain Scores were higher in CFS+MA than CFS+MO. These queries relied on self-reported perceptions of dizziness and lightheadedness, peripheral numbness, thoracic sensations, and higher numbers of pain descriptors. It is not clear how these perceptions may be related to the phenomenon of aura. Vertigo has been associated with migraine as vertiginous migraines, but has not been definitively investigated for a relationship to aura [25,26]. The sensations of abnormal heart beat and palpitations may be related to autonomic dysregulation that is a common feature of CFS [27]. Chest pain was not related to coronary artery disease or angina as determined by history and physical examination, EKG's, and exercise stress tests in a portion of the subjects. Noncardiac chest pain with perceptions of tachycardia and palpitations may be due to esophageal spasms with nutcracker esophagus [28] or costochondritis [29] in CFS.

Conversely, CFS+MO showed trends for an higher prevalence of FM (1990 criteria) [8] and lower systemic and sinus pain thresholds than CFS+MA (Table 5). Additional testing will be required to determine if these measures reach statistical significance when the number of CFS+MA subjects is increased. This potential difference in tenderness (hyperalgesia) and allodynia [23] between CFS+MA and CFS+MO was not apparent when the severities of the pain - related symptoms such as myalgia and arthralgia were compared (Figure 1). The severities of neurocognitive symptoms were also equivalent between CFS+MO and CFS+MA subgroups.

The current study was not designed to correlate triggers of migraine or the presence of an aura with precipitating factors of severe episodes of CFS complaints. Migraine triggers include fasting, premenstrual period hormonal status, lack of sleep, nasal irritants and odors [30]. Similar triggers are associated with acute worsening of CFS complaints. Future studies will be needed to identify correlates and mechanisms of aura, allodynia and hyperalgesia development in CFS migraineurs [16,20,23].

The association of migraine with CFS and FM introduces a new perspective for "functional" somatization disorders. We agree with Wessely et al. [31] who stated that the existence of specific functional somatic syndromes is an artifact of medical specialization and different systems - oriented consensus definitions of these illnesses. This attitude is conveyed by the 2010 FM diagnostic criteria that overlap extensively with CFS, and remove the importance of "tender points" [1,9]. A further stage in the evolution of CFS, FM, irritable bowel syndrome, and other systems - based diagnoses has been proposed by Fink and Schroder with their hypothesis of Bodily Distress Syndrome [32]. A reevaluation of the pathophysiological mechanisms contributing to these seemingly disparate syndromes and individual organic disorders such as migraine may lead to the recognition of common primary pathologic, genetic and environmental diatheses that lead to overlapping and fluctuating patterns of organ - specific complaints.

## Conclusions

CFS subjects have a high prevalence of migraine headaches that may be overlooked and undertreated. The proportion with an aura was similar to other migraine groups. CFS+MA was associated with higher severity scores for neural problems such as numbness and dizziness, and alterations of heart beat. The lower pressure - induced pain thresholds and hyperalgesia found in the CFS+MO subgroup was suggestive of nociceptive hyperresponsiveness and central sensitization. Appropriate diagnosis and treatment with triptans may be beneficial for CFS subjects and their complex headaches.

## Abbreviations

IHS: International Headache Society; CFS: Chronic Fatigue Syndrome; FM: Fibromyalgia; ACR: American College of Rheumatology; MA: Migraine with aura; MO: Migraine without aura; WPI: Widespread Pain Index; WSP: Widespread Pain; MRI: Magnetic Resonance Imaging; CSD: Cortical Spreading Depression; CSMI: Multisystem Complaints Questionnaire; MFI: Multidimensional Fatigue Inventory; SF-36: Medical Outcomes Survey Short Form 36.

## Competing interests

The authors declare that they have no competing interests.

## Authors' contributions

MKR and JNB made the original clinical observations, and performed the study with the assistance of the other authors. All authors have read and approved the final manuscript.

## Support

This project was conducted through the Georgetown-Howard Universities Center for Clinical and Translational Science and supported by the National Institutes of Health National Center for Research Resources through Grant 1UL1RR031975; U.S. Public Health Service Award R01 ES015382 from the National Institute of Environmental and Health Science; and the Congressionally Directed Medical Research Program (CDMRP) Award W81XWH-07-1-0618.

## Pre-publication history

The pre-publication history for this paper can be accessed here:

http://www.biomedcentral.com/1471-2377/11/30/prepub
